# Effects of coagulation factor XIII on intestinal functional capillary density, leukocyte adherence and mesenteric plasma extravasation in experimental endotoxemia

**DOI:** 10.1186/cc3994

**Published:** 2006-02-13

**Authors:** Jürgen Birnbaum, Ortrud Vargas Hein, Carsten Lührs, Oskar Rückbeil, Claudia Spies, Sabine Ziemer, Matthias Gründling, Taras Usichenko, Konrad Meissner, Dragan Pavlovic, Wolfgang J Kox, Christian Lehmann

**Affiliations:** 1 Klinik für Anaesthesiologie und operative Intensivmedizin, Charité – Universitätsmedizin Berlin, Campus Charité Mitte, 10117 Berlin, Schumannstr., Germany; 2 Insitut für Laboratoriumsmedizin und Pathobiochemie, Charité – Universitätsmedizin Berlin, Campus Charité Mitte, 10117 Berlin, Schumannstr., Germany; 3 Klinik für Anästhesiologie und Intensivmedizin, Ernst-Moritz-Arndt-Universität Greifswald, 17489 Greifswald, F.-Loeffler-Str., Germany

## Abstract

**Introduction:**

The objective of this study was to determine the effects of the administration of the coagulation factor XIII (F XIII) on intestinal functional capillary density, leukocyte adherence and mesenteric plasma extravasation during experimental endotoxemia.

**Methods:**

In a prospective, randomized, controlled animal study 42 male Wistar rats were divided into three groups. Group 1 served as the control group. Groups 2 (lipopolysaccharide (LPS) group) and 3 (F XIII group) received endotoxin infusions (2.5 mg/kg/h for 2 hours). In group 3, 50 U/kg body weight F XIII was continuously administered during the first 30 minutes of endotoxemia. F XIII levels were measured in all animals. One half of the animals of each group were studied for intestinal functional capillary density (FCD) and leukocyte adherence on venular endothelium by intravital fluorescence microscopy (IVM). In the other half of each group, mesenteric plasma extravasation (FITC-albumin) was determined by IVM.

**Results:**

The F XIII level was significantly increased in the F XIII treatment group. In the LPS group, endotoxemia led to a significant reduction of mucosal FCD (-18.5%; *p *< 0.01 versus control group). F XIII administration in the F XIII group attenuated the decrease in mucosal FCD (-3.7% compared to control; *p *< 0.05 versus LPS group). During endotoxemia, a significant increase of leukocyte adherence at the endothelium could be noted in the LPS group compared to the control group. Leukocyte adherence at the endothelium and plasma extravasation in the F XIII group did not differ significantly from the LPS group.

**Conclusion:**

Factor XIII protected mucosal capillary perfusion against endotoxin-induced impairment in an experimental sepsis model in rats, whereas leukocyte adherence and plasma extravasation remained unchanged.

## Introduction

Disturbance of endothelial integrity, especially in the gastrointestinal tract, is the hallmark of sepsis and septic shock. Poor perfusion of mucosal layers causes damage to the mucosal barrier and translocation of bacteria and their toxins into the systemic circulation [1,2]. Increased paracellular permeability leads to extravasation of fluids and macromolecules such as albumin into the intercellular space. The resulting edema causes disturbances of microcirculation and organ function or even organ failure. Edema formation is, therefore, a diagnostic criteria for sepsis [3] and also a main pathomechanism for the development of multiple organ dysfunction syndrome and multiple organ failure.

Being the last step in the coagulation cascade and in the regulation of fibrinolysis, coagulation factor XIII (F XIII) plays a vital role as a fibrin stabilizing agent in fibrin clot formation. The active form of F XIII (F XIIIa) is a transglutaminase that builds cross-links between polypeptide chains, thus protecting fibrin from fibrinolytic enzymes[4]. F XIII may have multiple effects in addition to its effects in the coagulation system: it plays a role in cell adhesion and migration [5], prevents edema formation due to its influence on endothelial barrier function [6-8], influences constitution of cellular and extracellular matrix [9,10] and it seems to promote wound and bone healing [11-13]. Cytoplasmatic expression of the F XIIIa subunit in macrophages is related very closely to phagocytic activities and thus to leukocyte activation [14].

Some data suggest that F XIII also has an influence on the integrity of gut mucosa that has changed because of inflammation [15-17]. During sepsis and septic shock, plasma levels of F XIII can decrease [18,19] and high levels of tumor necrosis factor alpha are found [20]. Tumor necrosis factor alpha activates neutrophil granulocytes [21] and thus is able to induce secretion of lysosomal enzymes, such as human neutrophil elastase. Endothelial cell monolayer permeability is increased by these enzymes [22]. Moreover, human neutrophil elastase degrades F XIII and can encourage hemostatic disturbances and organ dysfunction [23]. Low F XIII activity in sepsis is associated with the severity of illness and organ damage [19].

In the treatment of sepsis, F XIII may have several potentially beneficial effects due to the inhibition of leukocyte activation, cell adhesion and migration, as well as by diminishing the magnitude of plasma extravasation and protecting the gut mucosal integrity, perfusion and function. We put this hypothesis to the test by investigating the effect of F XIII on intestinal functional capillary density (FCD), leukocyte adherence on venular endothelium as a parameter of leukocyte activation and mesenteric plasma extravasation of fluorescein isothiocyanate (FITC) labeled albumin by intravital fluorescence microscopy (IVM) in experimental endotoxemia.

## Methods

### Animals

Forty-two male Wistar rats (200 to 250 g, 6 to 8 weeks old) were obtained from Tierzucht Schönwalde GmbH, Schönwalde, Germany, housed in chip-bedded cages in an air-conditioned animal quarter, and acclimatized for one week to the institutional animal care unit prior to the experiments. The animals were kept on a 12 hours light/dark cycle with free access to water (drinking bottle) and standard rat chow (Altromin^®^, Lage, Germany). Eighteen hours prior to each experiment food was withdrawn; water remained accessible. The animal experiments were approved by the Institutional Review Board for the care of animal subjects (protocol G 0133/00) and performed in accordance with German legislation on the protection of animals.

### Anesthesia and monitoring

The animals were initially anesthetized with 60 mg/kg pentobarbital (Sigma, Deisenhofen, Germany) intraperitoneally and were supplemented with 20 mg/kg/h pentobarbital intravenously in the course of the experiment. Fixation of the animals was carried out in supine position on a heating pad, keeping a rectal body temperature between 36.5°C (97.7°F) and 37°C (98.6°F). Tracheostomy was performed to maintain airway patency and animals breathed room air spontaneously. The left jugular vein and carotid artery were cannulated with polyethylene catheters (PE50; inner diameter 0.58 mm; outer diameter 0.96 mm; Portex, Hythe, Kent, UK). Arterial pressure and heart rate were recorded continuously (Biomonitor BMT 5231, RFT, Stassfurt, Germany). The animals received 7.5 ml/kg/h crystalloid solution (Thomaejonin^®^, Thomae, Biberach, Germany).

### General protocol

Experiments started 30 minutes after cannulation (time = 0 h). The rats were divided into 3 groups of 14 animals each. One half of the animals in each group underwent an examination of the submucosa for leukocyte adherence on venular endothelium and functional capillary density (FCD) by intravital fluorescence microscopy (IVM) of the small bowel wall. In the other half of the animals, plasma extravasation in the mesentery was determined by IVM.

The animals of group 1 (control group) did not receive endotoxin. In group 2 (lipopolysaccharide (LPS) group) and group 3 (F XIII group), endotoxemia (endotoxin challenge) was induced by continuous infusion of 2.5 mg/kg/h LPS from *Escherichia coli*, serotype O55:B5 (Sigma) over 2 hours. The animals of the control group were given an equivalent volume of normal saline (placebo infusion). In the F XIII group we continuously administered 50 U/kg body weight human F XIII (Fibrogammin^® ^HS, Aventis Behring, Marburg, Germany) during the first 30 minutes of endotoxemia.

Blood samples (total volume 0.2 ml) were taken 30 minutes after cannulation (time = 0 h) for white blood cell count determination as well as three hours after the endotoxin challenge (cell counter, Technicon H1, Bayer, Leverkusen, Germany). The F XIII levels were estimated at baseline (0 h) as well as 1.5 hours and 3 hours after the start of the endotoxin challenge.

Laparotomy for IVM was performed before the start of the endotoxin or placebo infusion. The abdomen was opened by a midline incision. A section of the distal small intestine orally from the ileocoecal valve was placed carefully on a specially designed stage attached to the microscope. During the entire *in vivo *microscopic procedure, the intestine was superfused with thermostat-controlled (37°C/98.6°F) crystalloid solution (Thomaejonin^®^) to avoid drying and exposure to ambient air [24]. The duration of each experiment, including induction of anesthesia, did not exceed 240 minutes. At the end of the experiments, the animals were euthanized by pentobarbital overdose.

### Intravital microscopy

IVM was performed using an epifluorescent microscope (Axiotech Vario, filter block No. 20, Zeiss, Oberkochen, Germany) with a 50-W HBO (Osram, Munich, Germany) short arc mercury lamp and equipped with a 10 × long distance (10/0.5; Fluar, Zeiss) and a 20 × water immersion (20/0.5; Achroplan, Zeiss) objective (mesentery: 40 × water immersion, 40/0,8; Achroplan, Zeiss) and a 10 × eyepiece. The images were transferred to a monitor (LDH 2106/00, Philips Electronics, Eindhoven, The Netherlands) by means of a video camera (FK 6990-IQ, Pieper, Schwerte, Germany) and were recorded on video at the same time using a video cassette recorder (Panasonic AG 6200, Matsushita, Japan) for off-line evaluation.

### Functional capillary density

After two hours of endotoxemia, 50 mg/kg bw FITC-labeled BSA (Sigma) was administered intravenously to distinguish plasma from red blood cells (negative contrast). The assessment of FCD in the intestinal mucosa and the circular as well as the longitudinal muscle layer was performed by morphometric determination of the length of red blood cell perfused capillaries per area in accordance with the method of Schmid-Schönbein and colleagues [25]. Five separate fields were examined in each layer.

### Leukocyte-endothelial interaction

After two hours of endotoxemia, leukocytes were stained *in vivo *by intravenous injection of 0.2 ml of 0.017 g % rhodamine 6G (MW 479; Sigma) for contrast enhancement, enabling visualization in the microvasculature. Microvessels in the intestinal submucosal layer were classified by their order of branching according to Gore and Bohlen [26]. Submucosal collecting venules (V 1) as well as postcapillary venules (V 3) were analyzed. The flux of rolling leukocytes was defined as the count of white cells moving at a velocity of less than two-fifths of that of erythrocytes in the centerline of the microvessels [27] and is quoted as non-adherent leukocytes passing through the observed vessel segment within 30 seconds. Adherent leukocytes (stickers) were defined in each vessel segment as cells that did not move or detach from the endothelial lining within an observation period of 30 seconds, and are quoted as the number of cells per mm^2 ^of endothelial surface, calculated from diameter and length of the vessel segment studied, assuming cylindrical geometry [27]. Seven vessels of each population were evaluated in every animal. The evaluation of leukocyte adherence was performed in a blinded fashion. The values were adjusted to the white blood cell count.

### Plasma extravasation

To quantify the plasma extravasation across mesenteric venules, 50 mg/kg bw FITC-BSA (Sigma) was injected 15 minutes before each experiment. The recorded fluorescent images were digitized and the gray levels were measured within five segments of the venule under study (Iv) and in five contiguous areas of the perivenular interstitium (Ip) depending on the fluorescence activity (gray levels range from 0 (black) to 255 (white)). Plasma extravasation (macromolecular leakage) was expressed as the ratio of Ip/Iv after one hour of endotoxemia. Evaluation was performed in a blinded fashion.

### Statistical analysis

Data analysis was performed using a statistical software package (SigmaStat, Jandel Scientific, Erkrath, Germany). All data were expressed as group mean ± standard deviation or standard error of mean and analyzed using a one-way analysis of variance followed by the Bonferroni corrected *t *test. Plasma extravasation, mean arterial pressure, heart rate and white blood cell count were analyzed by a two-way analysis of variance (repeated measures in the factor of time). This test was followed by the Scheffé test. A *p *value <0.05 was considered significant.

## Results

Hemodynamic changes that occurred in the macrocirculation are given in Tables 1 and 2. Blood pressure and heart rate remained stable in the control group. The endotoxin challenge resulted in a significant fall in mean arterial pressure in the LPS and the F XIII groups after one hour. The transient pressure drop was followed by stabilization in these two endotoxemic groups two hours after LPS administration. Two hours after the endotoxin challenge there was no difference in arterial pressure values in either endotoxemic group compared to the control group. The heart rate was increased significantly in both endotoxemic groups at this point in time.

After three hours the white blood cell count in the control group was 7.8 ± 1.6 × 10^3^/mm^3^. At this time point, the white blood cell count was significantly lower in the LPS and F XIII groups compared to the controls (LPS group, 3.0 ± 0.8 × 10^3^/mm^3^; F XIII group, 3.7 ± 1.0 × 10^3^/mm^3^; *p *< 0.05 both groups versus control group).

**Figure 1:**
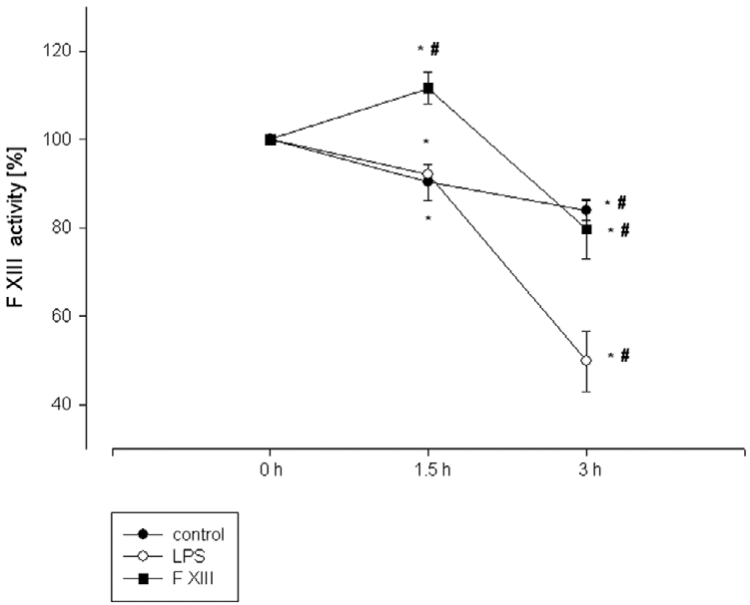
Factor XIII activity (percentage of baseline values, mean ± standard error of the mean; *p < 0.05 compared to baseline; ^#^*p *< 0.05 compared to lipopolysaccharide (LPS) group). Control, control group; F XIII, factor XIII treated LPS group; LPS, untreated LPS group.

After three hours of endotoxemia, F XIII activity fell to 49.8 ± 6.8% of baseline activity in the LPS group. F XIII administration resulted in an increase of F XIII activity to 111.7 ± 3.7 % after 1.5 hours and after 3 hours of endotoxemia the F XIII group values were in the range of the controls (Figure 1).

**Figure 2:**
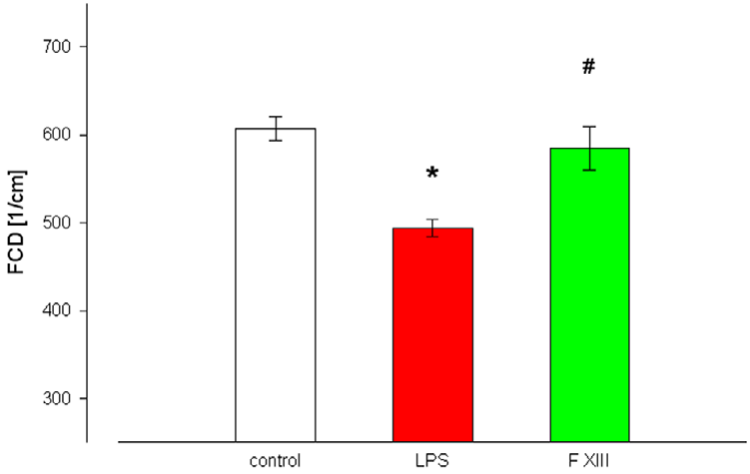
Effects of factor XIII administration on intestinal functional capillary density (FCD) in the mucosal layer during experimental endotoxemia. FCD after one hour of endotoxemia (mean ± standard error of the mean; *p < 0.05 compared to baseline; ^#^*p *< 0.05 compared to lipopolysaccharide (LPS) group). Control, control group, F XIII, factor XIII treated LPS group; LPS, untreated LPS group.

The changes in the FCD of the intestinal mucosa are shown in Figure 2. After two hours of endotoxemia we found a significant reduction of mucosal FCD in the LPS group (-18.5%; *p *< 0.01 versus control group). The F XIII administration attenuated the decrease in mucosal FCD (-3.7% compared to control; *p *< 0.05 versus LPS group). The FCD changes in the circular and the longitudinal muscle layers of the small intestine were not statistically significant (Table 3).

In the control group we determined a remarkable baseline rolling of the leukocytes along the endothelial lining of collecting (V 1) and postcapillary (V 3) venules. A significant decrease in the flux of rolling leukocytes was found in both the LPS and the F XIII groups (Table 4).

**Figure 3:**
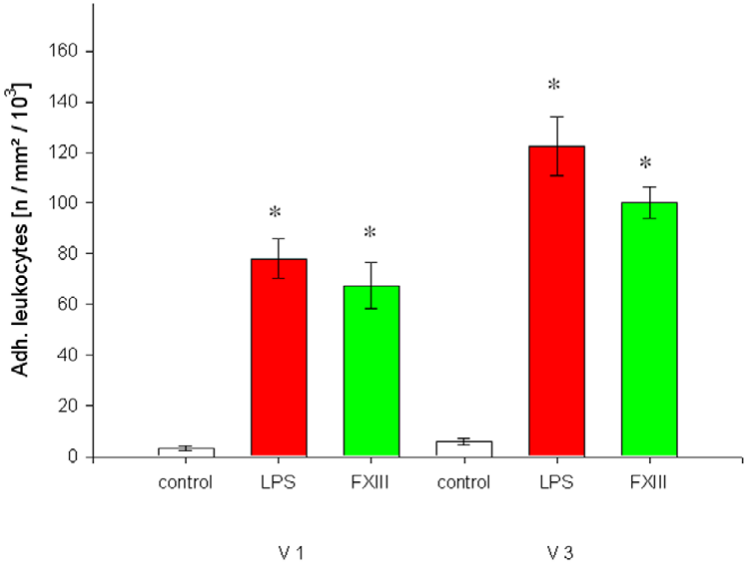
Effects of factor XIII administration on intestinal leukocyte adherence in the submucosal layer during experimental endotoxemia. Adherent (Adh) leukocytes in V1-/V3-venules after two hours of endotoxemia (mean ± standard error of the mean; *p < 0.05 compared to control group; *p *> 0.05 lipopolysaccharide (LPS) plus F XIII group compared to LPS group). Control, control group; F XIII, factor XIII treated LPS group; LPS, untreated LPS group.

Figure 3 illustrates the count of firmly adherent leukocytes two hours after start of the endotoxin challenge. In the LPS group a 24-fold increase was noticed in the count of sticking leukocytes in the collecting venules (V 1) compared to the control animals (*p *< 0.05). In postcapillary venules (V3) the increase was 20-fold (*p *< 0.05). This increase of leukocyte adherence was not influenced by the F XIII treatment in the FXIII group.

**Figure 4:**
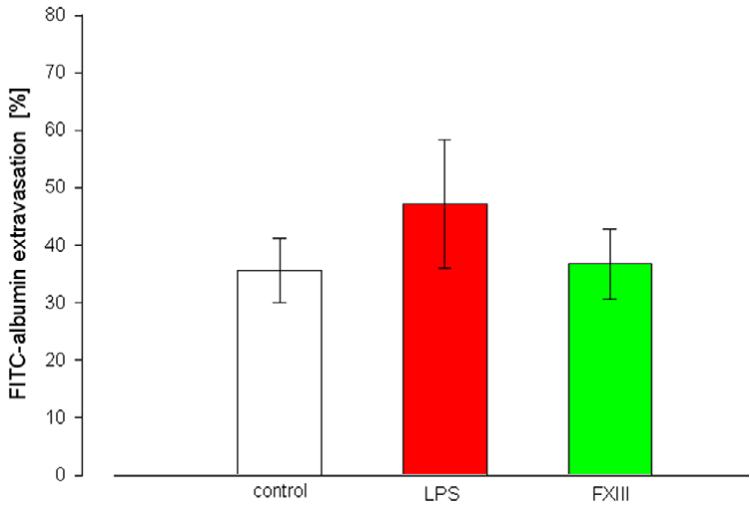
Effects of factor XIII administration on mesenteric plasma extravasation during experimental endotoxemia. Plasma extravasation after one hour of endotoxemia (mean ± SEM). Control, control group; F XIII, factor XIII treated LPS group; FITC, fluorescein isothiocyanate; LPS, untreated LPS group.

Figure 4 depicts plasma extravasation measured by FITC-BSA leakage across the venular endothelium in the mesentery after one hour of endotoxemia. During the experiment we observed a tendency for FITC-BSA extravasation to increase. There was no statistically significant difference between the groups.

## Discussion

The administration of F XIII in septic animals prevented the decrease of mucosal FCD as seen in the untreated LPS group compared to controls. We could not detect an influence of F XIII administration on leukocyte to endothelium interaction and plasma extravasation. After three hours, endotoxemia resulted in an expected decrease in F XIII activity compared to the control group. The decrease of F XIII in our sepsis model correlates with findings of clinical investigations in septic patients [18,19]. However, through F XIII substitution in the treatment group we achieved F XIII levels comparable to controls after three hours of endotoxemia.

In our study we found a higher FCD in the F XIII group compared to untreated endotoxemic animals, indicating that perfusion in capillaries of the intestinal mucosa was protected by F XIII administration. It appears that not only the preservation of microcirculation but also pro-angiogenic properties are related to the effects of factor XIII. *In vitro *F XIII caused a dose-dependent enhancement of array formation in a Matrigel tube formation model, while in a rabbit cornea model, injection of F XIII led to neovascularization [28]. As these effects occur over rather a long time (measured after 16 or 48 hours, respectively), the preservation of perfusion in the capillaries in our study can not be explained by these mechanisms. F XIII plays an important role in the final stage of the coagulation cascade, cross-linking fibrin monomers and stabilizing the fibrin clot. Thus, even an impairment of capillary perfusion as a result of augmented fibrin clot formation after the administration of this procoagulative substance is conceivable. In a sepsis model in rabbits it was shown that F XIII plays a role in promoting LPS induced disseminated intravascular coagulation with resulting organ damage [29]. In our study, however, we saw an increase in functional perfused capillaries in the gut mucosa.

Perfusion in the capillaries could be impaired directly by the formation of tissue edema. This is why FCD should also be discussed in the context of plasma extravasation with consecutive edema formation. In our study endotoxemia did not result in the expected increased mesenteric plasma extravasation. We found that the LPS group tended to have higher plasma extravasation, although this difference was not statistically significant between the groups. Due to the high discrepancy in plasma extravasation values, a significant difference between the groups could be expected after testing a greater number of animals.

A protective effect of F XIII on the endothelial barrier function was shown in several other studies [6-8,30-32]. Hirahara and colleagues [32] investigated the effect of F XIII on permeability in guinea pig endothelial cells, enhanced by intradermal injection of anti-endothelial cell antiserum administered into the dorsal skin. Antiserum or a mixture of antiserum and F XIII was injected after Evans blue injection, and later the quantity of Evans blue was determined at each injection site. F XIII had a suppressive effect on dye leakage and on swelling induced by the antiserum. The authors assumed that F XIII plays an important role in inflammatory sites and that it may act as an anti-inflammatory protein. Due to its anti-inflammatory effects, F XIII also might contribute to preservation of perfusion in the capillaries. In a model of cultured monolayers of porcine aortic endothelial cells and in saline-perfused rat hearts, F XIII reduced the albumin permeability of endothelial monolayers [6]. The increase in myocardial water content in ischemic-reperfused rat hearts was prevented, indicating that activated F XIII reduces endothelial permeability [6].

The protective effect of F XIII on vascular endothelium integrity has also been documented in clinical investigations. In a prospective investigation of perioperative cardiac edema formation requiring a delayed sternal closure in children, F XIII or placebo was substituted preoperatively. The substitution of F XIII reduced the incidence of myocardial swelling and the authors concluded that the clinical application of F XIII may have a valuable therapeutic benefit in cases of leakage syndrome during extracorporeal circulation in congenital heart surgery [8].

F XIII administration in our model tended only to lower plasma extravasation in comparison to the endotoxin group and this was not statistically significant for mesenteric venules.

After administration of F XIII, a tendency to attenuate the leukocyte adherence could be noticed in our study. Tissue transglutaminase and F XIIIa are expressed on the surface of monocytic cells and some evidence indicates the involvement of transglutaminases in cell adhesion, for example, they can influence adhesion of monocytic cells on fibronectin [33]. Factor XIIIa promoted adhesion and spreading of different cells, such as human liver cells, human leukemia cells, human melanoma cells and bovine aortic endothelial cells, to F XIIIa coated surfaces *in vitro *[5], indicating that F XIIIa itself mediates cell adhesion. In an adhesion assay, it was shown *in vitro *that F XIIIa mediates adhesion of the human microvascular endothelial cell line HMEC-1 and that F XIIIa binds to HMEC-1 cells in solution. This has been verified by a flow cytometric analysis [34].

Integrins as transmembrane receptors mediating cell-cell interactions are expressed on leukocytes. They play an important role in leukocyte transendothelial migration. Some integrins, especially a_4_ß_1 _and a_9_ß_1 _integrins, are ligands for F XIII [35]. Taking these findings into consideration, as well as the above mentioned sealing effect of F XIII, enhancement of the interaction between leukocytes and the vascular endothelium by F XIII could be expected. The role of F XIII in relation to the interaction of leukocytes to the vascular endothelium, however, requires further investigation.

## Conclusion

Factor XIII can protect mucosal capillary perfusion against endotoxin-induced impairment in an experimental sepsis model in rats. Because of the importance of preservation of intestinal perfusion in the early treatment of sepsis and septic shock, early F XIII administration might be considered in septic patients but requires clinical approval.

## Key messages

• 	Factor XIII protected the perfusion in mucosal capillaries against endotoxin-induced impairment in an experimental sepsis model in rats.

• 	In septic patients, F XIII administration might be favourable but requires clinical approval.

## Abbreviations

BSA = bovine serum albumin; F XIII = factor XIII; FCD = functional capillary density; FITC = fluorescein isothiocyanate; IVM = intravital fluorescence microscopy; LPS = lipopolysaccharide.

## Competing interests

The authors declare that they have no competing interests.

## Authors' contributions

JB and OVH coordinated the study and drafted the manuscript. CL, OR, MG, TU, KM and DP performed the IVM, collected the data and helped to draft the manuscript. SZ performed the estimation of F XIII levels and helped to draft the manuscript. CS, WJK and ChL conceived and designed the study and performed the statistical analysis.

## Figures and Tables

**Table 1:** Mean arterial pressure (mmHg)

Group	Time		
	0 h	1 h	2 h
Control	107 ± 14	98 ± 11	115 ± 15
LPS	109 ± 19	90 ± 9^a^	108 ± 4
F XIII	123 ± 15	94 ± 14^a^	107 ± 6

Values are mean ± standard deviation. ^a^*p *< 0.05 compared to baseline. Control, control group; F XIII, factor XIII treated lipopolysacharide (LPS) group; LPS, untreated LPS group.

**Table 2:** Heart rate (beats/min)

Group	Time		
	0 h	1 h	2 h
Control	344 ± 28	340 ± 20	341 ± 22
LPS	367 ± 17	344 ± 24	379 ± 36^a^
F XIII	373 ± 26	370 ± 23	386 ± 13^a^

Values are mean ± standard deviation. ^a^*p *< 0.05 compared to control group. Control, control group; F XIII, factor XIII treated lipopolysacharide (LPS) group; LPS, untreated LPS group.

**Table 3:** Functional capillary density in the intestinal longitudinal and circular muscle layer (1/cm)

Muscle	Group		
	Control	LPS	F XIII

Longitudinal	202.6 ± 14.0	199.3 ± 23.1	200.8 ± 33.0
Circular	208.7 ± 26.1	235.3 ± 22.3	249.3 ± 46.1

Values are mean ± standard deviation. Control, control group; F XIII, factor XIII treated lipopolysacharide (LPS) group; LPS, untreated LPS group.

**Table 4:** Flux of rolling leukocytes in V1/V3 venules (n/min)

Venules	Group		
	Control	LPS	F XIII

V1	35.8 ± 25.6	2.5 ± 1.7^a^	4.4 ± 2.9^a^
V3	29.5 ± 20.1	1.8 ± 1.0^a^	2.7 ± 1.8^a^

Values are mean ± standard deviation. ^a^*p *< 0.05 compared to control group. Control, control group; F XIII, factor XIII treated lipopolysacharide (LPS) group; LPS, untreated LPS group; V1, submucosal collecting venules; V3, postcapillary venules.
